# Data Collection and Management of mHealth, Wearables, and Internet of Things in Digital Behavioral Health Interventions With the Awesome Data Acquisition Method (ADAM): Development of a Novel Informatics Architecture

**DOI:** 10.2196/50043

**Published:** 2024-08-07

**Authors:** I Wayan Pulantara, Yuhan Wang, Lora E Burke, Susan M Sereika, Zhadyra Bizhanova, Jacob K Kariuki, Jessica Cheng, Britney Beatrice, India Loar, Maribel Cedillo, Molly B Conroy, Bambang Parmanto

**Affiliations:** 1 School of Health and Rehabilitation Science, University of Pittsburgh, Pittsburgh, PA, United States; 2 School of Nursing, University of Pittsburgh, Pittsburgh, PA, United States; 3 School of Public Health, University of Pittsburgh, Pittsburgh, PA, United States; 4 Nell Hodgson Woodruff School of Nursing, Emory University, Atlanta, GA, United States; 5 School of Medicine, University of Utah, Salt Lake City, UT, United States

**Keywords:** integrated system, IoT integration, wearable, mHealth Fitbit, Nokia, clinical trial management, research study management, study tracking, remote assessment, tracking, Fitbit, wearable devices, device, management, data analysis, behavioral, data collection, Internet of Things, IoT, mHealth, mobile health

## Abstract

The integration of health and activity data from various wearable devices into research studies presents technical and operational challenges. The Awesome Data Acquisition Method (ADAM) is a versatile, web-based system that was designed for integrating data from various sources and managing a large-scale multiphase research study. As a data collecting system, ADAM allows real-time data collection from wearable devices through the device’s application programmable interface and the mobile app’s adaptive real-time questionnaires. As a clinical trial management system, ADAM integrates clinical trial management processes and efficiently supports recruitment, screening, randomization, data tracking, data reporting, and data analysis during the entire research study process. We used a behavioral weight-loss intervention study (SMARTER trial) as a test case to evaluate the ADAM system. SMARTER was a randomized controlled trial that screened 1741 participants and enrolled 502 adults. As a result, the ADAM system was efficiently and successfully deployed to organize and manage the SMARTER trial. Moreover, with its versatile integration capability, the ADAM system made the necessary switch to fully remote assessments and tracking that are performed seamlessly and promptly when the COVID-19 pandemic ceased in-person contact. The remote-native features afforded by the ADAM system minimized the effects of the COVID-19 lockdown on the SMARTER trial. The success of SMARTER proved the comprehensiveness and efficiency of the ADAM system. Moreover, ADAM was designed to be generalizable and scalable to fit other studies with minimal editing, redevelopment, and customization. The ADAM system can benefit various behavioral interventions and different populations.

## Introduction

In recent years, the health care industry has focused a considerable amount of attention on wearable devices because of their capabilities in monitoring and collecting clinically relevant information such as heart rate (HR), blood pressure, respiratory rate, sleep, and physical activity [1]. One of the main drivers of this increased attention is the high level of adoption of wearable devices in the United States. Among all US adults, around 30% have experience with wearable devices and nearly half of those who use their wearable devices regularly are willing to share their data for research purposes [2]. Furthermore, there is an increase in the accuracy offered by these off-the-shelf wearables in providing health-related measurements. For example, several studies have been conducted on consumer wearable devices such as the Apple Watch, Samsung Gear, Fitbit, Huawei Band, and Xiaomi Mi Band, which reported high accuracy in measuring steps, distance, and HR [3-6]. These trends and the maturity of consumer wearable technologies have led more researchers to use consumer wearable devices in their research studies.

However, this increasing use of wearable devices in research studies also presents challenges. Managing and analyzing the large amounts of data generated by wearable devices in research studies in real time is not a simple task, especially for research teams without dedicated specialized technical support in wearable data management and data engineering. For example, the Fitbit fitness band, one of the most widely used consumer wearable devices in research, has been used to track steps [4,5], physical activity [4-14], HR [15], and sleep patterns [16]. Among the publications we reviewed (n=15), many (n=6, 40%) of the studies using the Fitbit fitness band used a third-party, proprietary, web-based Fitbit data collection and visualization platform—Fitabase [6-9,11,17]; 3 (20%) studies reported that they manually checked and downloaded participants’ data from participants’ individual Fitbit account at the end of the study [5,12,17]; 1 (7%) study used the MySantéMobile system [13]; and the rest (n=5, 33%) did not report on the system they used for collecting and displaying data from the wearable devices [4,10,14-16]. At the time of writing, Fitabase is the most popular data collection system, having been used in more than 450 institutions and more than 1100 studies [18]. The advantage of using Fitabase is that it can automatically collect data for all participants in real time and provides a basic dashboard function for individual users. However, Fitabase can only access data from Fitbit-related products, and the dashboard cannot be adapted to study-specific needs. For example, although Nokia is one of the widely used personal weight scales in weight-related research studies [19-24], it is not supported by Fitabase. The lack of a data collection and management system that is useful across wearable technologies is a major barrier for researchers who would like to use multiple wearable devices in a study.

Mobile health (mHealth) methods use advanced interactive technologies for the assessment of behaviors and delivery of digital health interventions, an area of research that is growing rapidly. Typical mHealth systems [25-31] consist of a smartphone app for data collection and intervention delivery, sensors integration, and occasionally clinical or administrative portals. However, although mHealth approaches may be readily adopted in behavioral research, it does not encompass end-to-end research management functions such as participant recruitment, screening, randomization, and retention.

A comprehensive and integrated implementation of a clinical trial management system in addition to wearable and mHealth integrations would allow the tracking of the study based on its specified recruitment needs, guarantee that the study starts and ends on time, make sure the necessary action has been taken at participants’ specialized milestones, and minimize study costs [32]. However, disjointed implementations and noninteroperable third-party systems have built inseparable barriers for researchers to take advantage of valuable data and track their study process efficiently. The three main barriers are as follows: (1) integrating data from various sources into 1 database poses difficulties [4-6,11,17]; (2) data are seldom ready to be processed for study-specific needs in real time, with previous attempts seeing varying degrees of success [12,14-17,19-24]; and (3) no end-to-end study management systems that address (1) and (2) are readily available on the market. Therefore, we designed and implemented the Awesome Data Acquisition Method (ADAM) system to address these barriers by providing a digital platform that enabled the management and collection of long-term health data from a variety of the Internet of Things (IoT) and wearable devices in an accurately and timely manner, thus allowing an accurate display of tailored and aggregated data in real time. The ADAM system significantly reduced data integration burdens, that is, (1) by having the most common third-party application programming interface (API) integration templates readily available and (2) saving costs in research management areas, such as recruitment, screening, enrollment, assessment scheduling, participant tracking, and recruitment reporting, by streamlining and digitalizing those processes, all of which support an efficient and rigorous study management experience.

## General Design

The Iterative and Incremental Development model [33] was used in the development of ADAM. In implementing the Iterative and Incremental Development model, the development team devised a road map outlining core functionalities essential for supporting capabilities that will be mentioned later. Subsequent iterations were planned based on participants’ and study coordinators’ (end-users’) feedback, as well as the latest technological advancements, with each iteration focusing on delivering a functional and deployable software increment. Criteria for transitioning from one increment to the next were determined by predefined milestones, including successful feature implementation, user experience enhancements, and adherence to performance metrics. End-user feedback played a pivotal role throughout the process, guiding feature enhancements and usability improvements, while rigorous testing procedures ensured the stability and reliability of each increment before release. This approach facilitated the creation of a responsive and user-centric software solution that continually evolved to meet user needs throughout the project.

The ADAM system was designed to support the entire management of research studies that include the use of commercial wearable devices and smartphone apps, including designing questionnaires and collecting the response data remotely, recruitment, screening, enrollment, data tracking, data monitoring, and data visualization. Those components were developed iteratively in each development cycle until the desired study flow was reached. The ADAM system consists of 3 main components: a mobile app, a portal, and a connection with the APIs of wearable devices.

## Architecture

**Figure 1.**
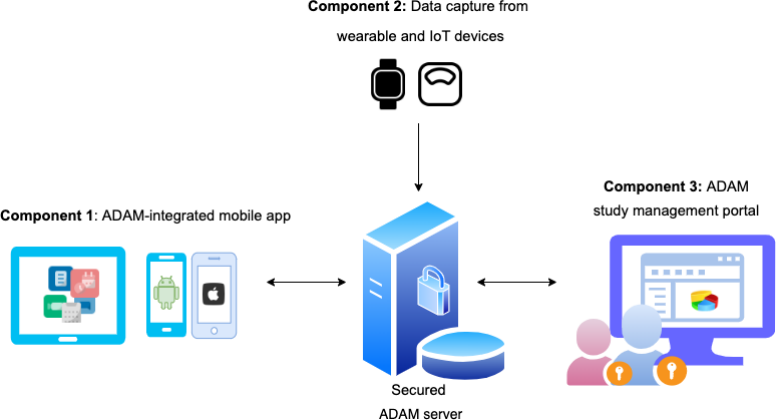
The architecture of the Awesome Data Acquisition Method (ADAM) system. IoT: Internet of Things.

The architecture for the ADAM system is illustrated in Figure 1. The 3 main components of ADAM include the ADAM-integrated mobile app, called the SMARTER app in the SMARTER trial, which is a cross-platform app that can run on Android and iOS devices (smartphones or tablets); a data capture component, which is used to capture and track the study-related data from the wearable and IoT devices for all participants; and the study management portal, which allows study coordinators to collect data and manage the entire study process. These components are connected to a secured ADAM server. We designed the ADAM server to have 1-way data communication with wearable device APIs and 2-way data communication between the ADAM-related mobile app and the clinical portal.

Figure 1 illustrates the architecture of ADAM (the overall system will be referred to as simply ADAM or the ADAM system, to differentiate from its components), which consists of components for data capture from commercial digital wearables and IoT devices through APIs. Currently, ADAM has implemented integration with open APIs from Fitbit and Nokia. Participants’ data were automatically collected from the wearable and IoT vendor’s servers regularly, with a frequency that was customized to the study. The data transfer between wearable devices, servers, and ADAM was secured through a unique token. Furthermore, all data at transit were secured via Transfer Layer Security (TLS/HTTPS). A similar method of pulling data from other off-the-shelf wearable devices could be used beyond the 2 vendors, Fitbit and Nokia.

We implemented ADAM as a system to support the SMARTER trial, which was conducted to evaluate the efficacy of 2 different approaches to a technology-supported (mHealth, wearable, and IoTs) behavioral intervention for weight loss [34-36]. The study used a randomized controlled trial design involving 502 adult participants who were randomized with equal allocation to 1 of 2 groups: the first group (n=250) received self-monitoring (SM) alone, while the second group (n=250) received remotely delivered feedback messages tailored to the recorded SM data. The main component of the SMARTER study intervention was personalized feedback messages via the ADAM-integrated SMARTER app, an investigator-developed mHealth app. The SMARTER app was connected to the ADAM portal through 2-way secure communications. Overall, ADAM, as a platform, was designed for the delivery of the study’s feedback intervention that included secure real-time messaging, adaptive questionnaires that could be delivered promptly, and reminders. As a study tool, we designed the ADAM system to track participants’ activities, such as the time when participants received or sent messages related to the study and the time when participants opened the app. All these data have been collected for future analysis.

Another component of the ADAM system was the ADAM portal, which includes a dashboard for study coordinators to review data and engage study participants. Study coordinators were able to get a bird’s-eye view of all participants in the study, including a map of their geographical locations. The coordinator was also able to view the detailed data of individual participants, including questionnaire data, screening data, randomization status, and real-time wearable device data. The ADAM portal consists of 8 modules: a wearable device module for pulling data from the Fitbit and Nokia servers to the ADAM server; a questionnaire module for designing and collecting questionnaires; a screening and enrollment module for tracking participants’ status in the study; a study phase–tracking module for providing a snapshot of the whole study process; a randomization module for study-customized randomization needs to help the interventionist performed the randomization at the baseline visit [34]; an assessment-scheduling module for remote or in-person assessment alerts and scheduling; a data-tracking and visualization module for reviewing participants’ data; and a retention management module for follow-up assessment data collection and remuneration management. All features worked closely with each other throughout the study data flow.

## Capabilities

### Customized Questionnaire Design and Remote Assessment Collection

Questionnaire-based assessment is one of the most common methods of data collection in research settings. Therefore, the ADAM portal was designed around this need, from the process of questionnaire and assessment design to the remote delivery of questionnaires. Designing a questionnaire based on the study needs was made simpler for researchers with the inclusion of an easy-to-use, drag-and-drop interface and a library of frequently used question templates. The adaptive questionnaire feature in ADAM was designed to be used by study coordinators and investigators with no need to do programming, with a similar ease of use to that of proprietary solutions such as Qualtrics, but unlike most of these proprietary solutions, ADAM allowed integrations with an mHealth app. The ADAM portal also supported more advanced questionnaire features such as input-type validation, error messages, required field validation, and question branching. After a questionnaire was published in the ADAM portal, depending on the purpose, a public link or a participant-specific private link was generated for study use. Once the participant had answered and submitted this questionnaire via the given link, the ADAM portal automatically formatted the answers, placed time stamps, calculated scores, and triggered any necessary flags and status updates that could be immediately reviewed by the study coordinators. As the ADAM portal was designed to support multiphase study needs, questionnaires could be generated for each study phase while considering different restrictions for each phase, for example, adding certain filtering restrictions in the screening phase for certain criteria in the screening phase’s questionnaire. This questionnaire module can also be adapted to fit the need for remote participant consent delivery, as the study consent form usually shares the same structure and data requirements as an assessment questionnaire.

### Study Management and Multiphase Study Tracking

The ADAM portal also supported the management of other steps in the research study, which commonly included multiple phases or processes such as recruitment, enrollment, engagement, and retention. For the recruitment phase, the ADAM portal supported a multistep screening process. Each sequential screening step could be triggered by the answers or the calculated scores of the current step’s screening or assessment questionnaire. It could also support manual evaluation by study coordinators. Once screening decisions had been made, the system automatically moved the eligible participants to the next phase in the ADAM system. This real-time process reduced the study coordinators’ manual data review and data calculation workload. It also helped reduce the study cost by improving the overall recruitment process’s efficiency.

For the enrollment and engagement phases, the ADAM portal has a built-in account management system that can assign a unique study-specific ID for each participant for efficient progress tracking while protecting their identifiable information. Furthermore, these IDs can also be used as common IDs linking data coming in from outside sources such as the Fitbit and Nokia APIs. With the benefit of the study-specific ID, the ADAM portal can easily record participants’ digital footprints within the system and track the milestones and progress of all participants within the study. The portal also provides the study coordinator with access to aggregated data, such as that of important milestones or sorted by group, which is represented in a CONSORT (Consolidated Standards of Reporting Trials) flow diagram. Such a flow diagram is valuable for study management in 3 aspects: it allows the study coordinator to switch the recruitment effort to a specific group of people during recruitment in cases where a group has a less-than-optimal recruitment rate; it facilitates the adjustment of the speed of recruitment to help make sure the study can start and end on time; and it can monitor the screening process and rate of attrition, which may provide some hints for adjustments that may be needed in screening methods.

For the retention process, the ADAM portal provides a dashboard for showing study-related alerts or flags and participants’ performance according to the data gathered from wearable devices, the digital scale, and the study app. Using this dashboard, the study coordinators have a near real-time report on whether participants are following the study protocol strictly, in other words, adhering to the study; as a result, they can intervene or take alleviating actions at a point closer to when participants began to show a trend toward lower adherence. This feature also helps to estimate the milestones for participants’ assessments. Other features are study compensation and remuneration management, which were considered indispensable parts of the ADAM system. Study coordinators used the remuneration feature of the ADAM portal to record all payments made or planned at different time points in the study.

### Data Synchronization With Wearable and IoT Devices

Data synchronization with wearable and IoT devices is one of the key features of the ADAM system. As more people rely on commercially available wearable and IoT devices to get their health data and track their fitness, a growing number of investigators have used this type of off-the-shelf wearable device as part of their studies. This trend indicates the need for a study management system that can integrate IoT and wearable data from multiple providers and vendors. Currently, the ADAM portal provides integration with Fitbit (wearable) and Nokia (IoT), since these companies have the largest market share, a secure authentication process, and stable open APIs. The steps to integrate data from wearables in ADAM from the users’ (study coordinators’) perspective are made simple. Study coordinators first need to create participant accounts in the ADAM portal and get participants’ permission and authorization to collect their data from outside APIs. Once ADAM has been granted authorization, data synchronization for the given participant will be scheduled daily. The ADAM portal also supports subscription-based data integration, where any new data that become available in the wearable will trigger synchronization or a data import into the ADAM database in a real-time fashion, although we did not implement this feature in the SMARTER study.

### Randomization

The ADAM system provided randomization by minimization, a more advanced randomization method, which was used to ensure the balance of prognostic factors between treatment groups and set equal treatment allocations [37].

### Real-Time Data Analysis for Study-Related Apps

ADAM adopts Firebase Analytics libraries to anonymously monitor app use and statistics. This part of the data mainly describe the use of the app within a period. The study team can obtain a real-time visualization on the ADAM main page. This information can provide an overview of the app use among all study participants and additional background information for the efficiency of message delivery.

## Evaluation of the ADAM Platform in the SMARTER Trial

### Overview

An evaluation of the ADAM platform was conducted as part of a weight-loss clinical trial called the SMARTER trial [34]. This study was registered on ClinicalTrials.gov (NCT033677936) and approved by the Institutional Review Board of the University of Pittsburgh. The SMARTER trial was a randomized controlled trial that followed a single-site, 2-group design, with a 1-year study duration for both comparison and intervention groups [34]. In the SMARTER trial, participants were allocated equally to the 2 treatment conditions: SM only or SM with real-time tailored feedback messages (SM+feedback) [34]. The study provided all participants with a Fitbit Charge 2 and a Nokia Scale for physical activity and weight SM; participants used the Fitbit app for dietary SM. The SMARTER app generated 3 tailored messages per day responding to the SM data provided, for example, diet, physical activity, and weight. More details for the SMARTER trial design and screening criteria can be found in the study design paper [34].

### Recruitment and Screening

**Figure 2.**
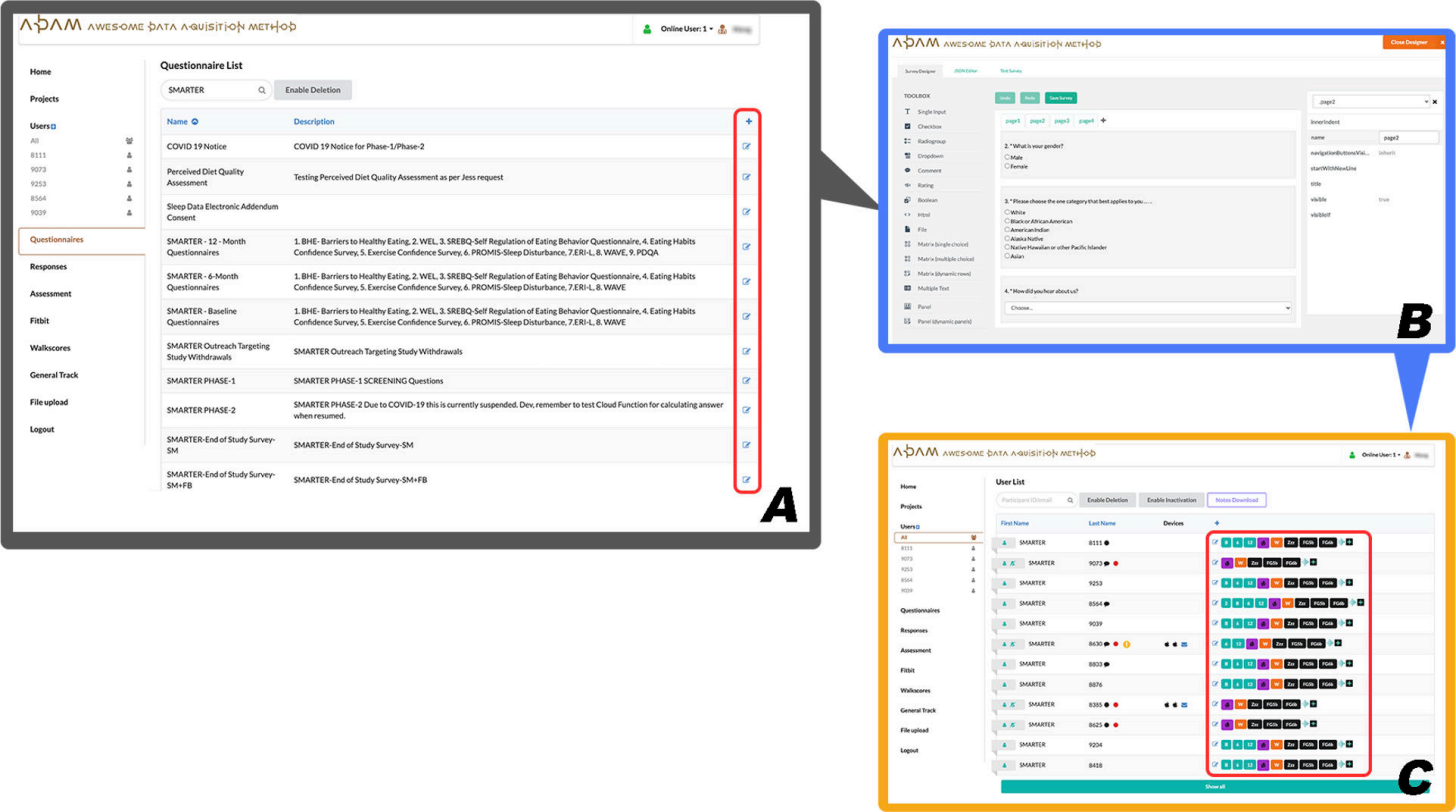
Customized questionnaire module of the ADAM portal: (A) questionnaire list, (B) edit or create a questionnaire, and (C) personal link for the questionnaire. ADAM: Awesome Data Acquisition Method.

**Figure 3.**
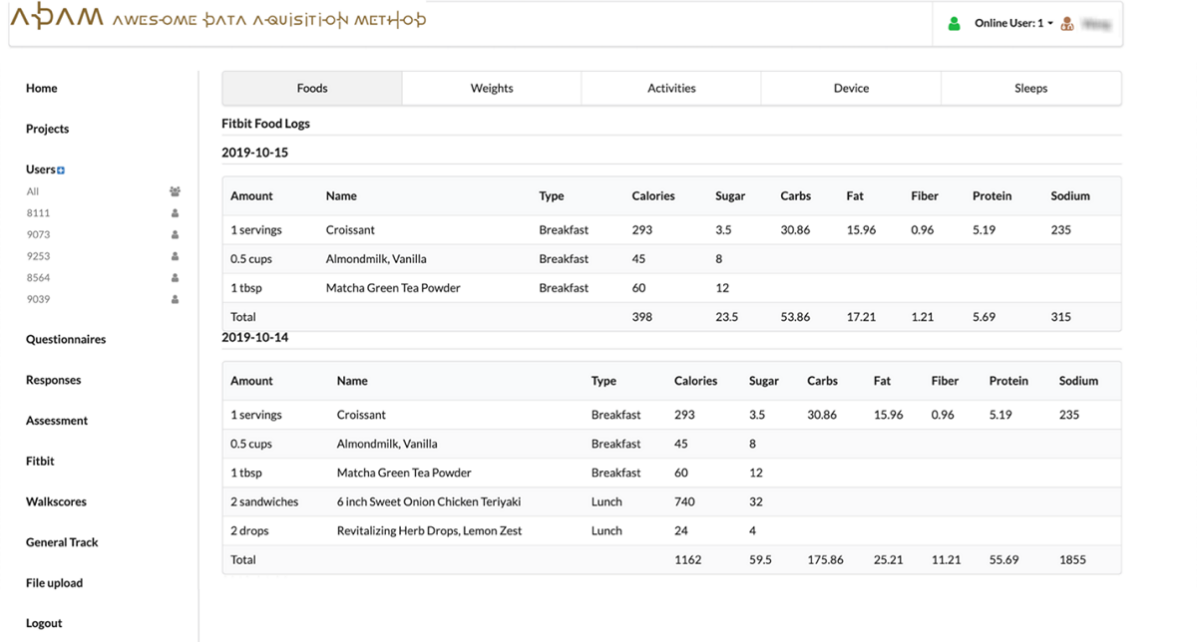
Food diary data collected from Fitbit. ADAM: Awesome Data Acquisition Method.

The SMARTER trial’s recruitment began in August 2018, and the trial itself was completed in March 2021. This study was designed to include a 3-phase screening process. The first 2 screenings were based on web-based questionnaires, where the ADAM portal provided autoscreening services based on the answers submitted by the participants. As shown in Figure 2, there were 3 steps for generating a questionnaire: Figure 2A lists all the questionnaires and consent forms from the SMARTER trial that were available on the ADAM portal; Figure 2B shows the straightforward drag-and-drop interface to design or edit a questionnaire; and Figure 2C shows that the ADAM portal could autocreate a specific questionnaire link for each participant on the user list page in the ADAM portal. In the SMARTER trial, phase-1 and phase-2 questionnaires were the study’s basic screening surveys, and the ADAM portal provided automatic screening of the candidates based on preset criteria such as aged ≥18 years, BMI between 27-43 kg/m^2^, smartphone user, general health history, lifestyle, and health and medical history. In phase 3, the ADAM portal still handled the display, submission, and scoring processes of the questionnaire but also gave the screening decisions back to the study coordinators. In this phase, participants were asked to complete a 5-day SM food diary using the Fitbit app. As illustrated in Figure 3, in our ADAM portal, the food diary data were automatically pulled from the Fitbit server for the study coordinators to review. Collecting data in one place relieved the pressure on study coordinators from switching back and forward between several different data management systems and tools.

### Randomization and Enrollment

**Figure 4.**
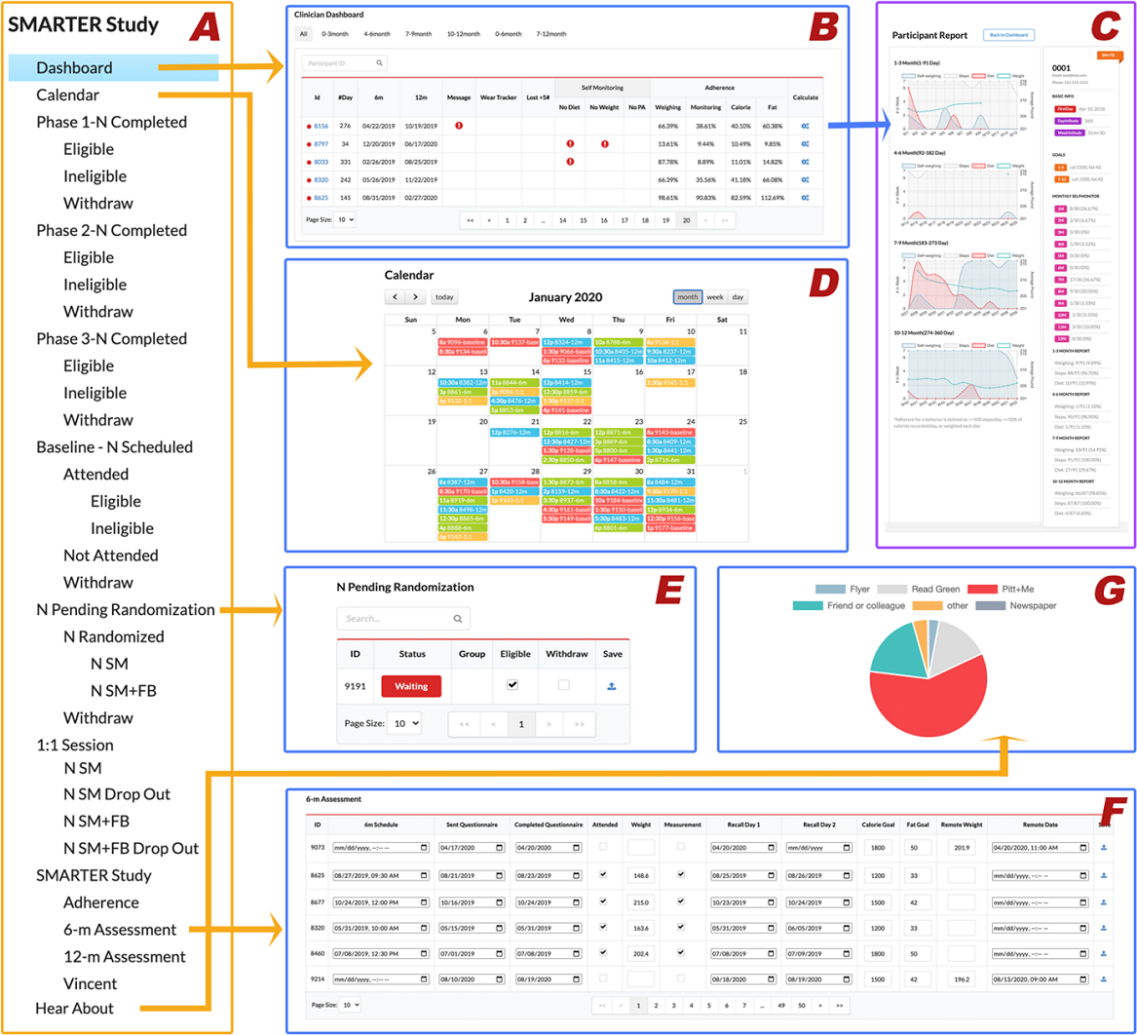
The general data-tracking module for SMARTER trial management in the ADAM portal: (A) recruitment steps, (B) clinician dashboard, (C) participant report, (D) calendar, (E) randomization page, (F) 6-month assessment page, and (G) referral breakdown. ADAM: Awesome Data Acquisition Method.

As displayed in Figure 4A, all the SMARTER trial recruitment steps were visualized and cataloged in the ADAM system. By clicking on each recruitment step, study coordinators could review the current remaining number of participants in that phase and make decisions on moving them to the next phase of the study. Once participants were ready for the randomization step, they would be displayed on the randomization page, as shown in Figure 4E. The ADAM system provided a minimization randomization algorithm to support this randomization process. After randomization, participants’ Fitbit and Nokia accounts were linked to our ADAM system by study coordinators; all the Fitbit-related and Nokia-related data including physical activity, food diary, and self-weighing data could then be collected by the ADAM system daily for the following 1-year intervention period.

### Engagement and Retention

After randomization, participants were asked to do daily SM during the 1-year study and complete 3 assessments at baseline, 6 months, and 12 months. During the 1-year study, participants were asked to adhere to the daily SM protocol by wearing a Fitbit tracker to record their daily physical activities, report food intake using the Fitbit app, and weigh themselves using a smart scale. All data were synchronized to the ADAM system and were displayed in a raw format, as shown in Figure 3, as well as a study-tailored precalculated report format, as shown in Figure 4B and C. The *clinical dashboard* in Figure 4B acts as a valuable resource for study coordinators to track participants’ adherence to all SM protocols and to receive alerts if participants lose weight too rapidly (a precaution to prevent unwanted clinical effects) or have poor study adherence. Study coordinators used this page to mark withdrawals at any time during their study period. The ADAM portal then automatically recorded and marked the withdrawal date appropriately. The trial included 3 assessments that heavily used the ADAM portal as a method for appointment scheduling and web-based survey completion. Figure 4F represents an example of a 6-month assessment page, and Figure 4D is an all-in-one assessment calendar. Due to the COVID-19 pandemic, in-person assessments were no longer permitted as of March 2020. The study team made the decision to change all the assessments to be conducted remotely and to obtain the primary outcome of weight directly from participants’ smart scales in their homes instead of the usual scale at the research center. Due to the remote-ready adaptive design of the ADAM system, this COVID-19–triggered transition was able to be performed quickly and with almost no interruption to the study flow.

**Figure 5.**
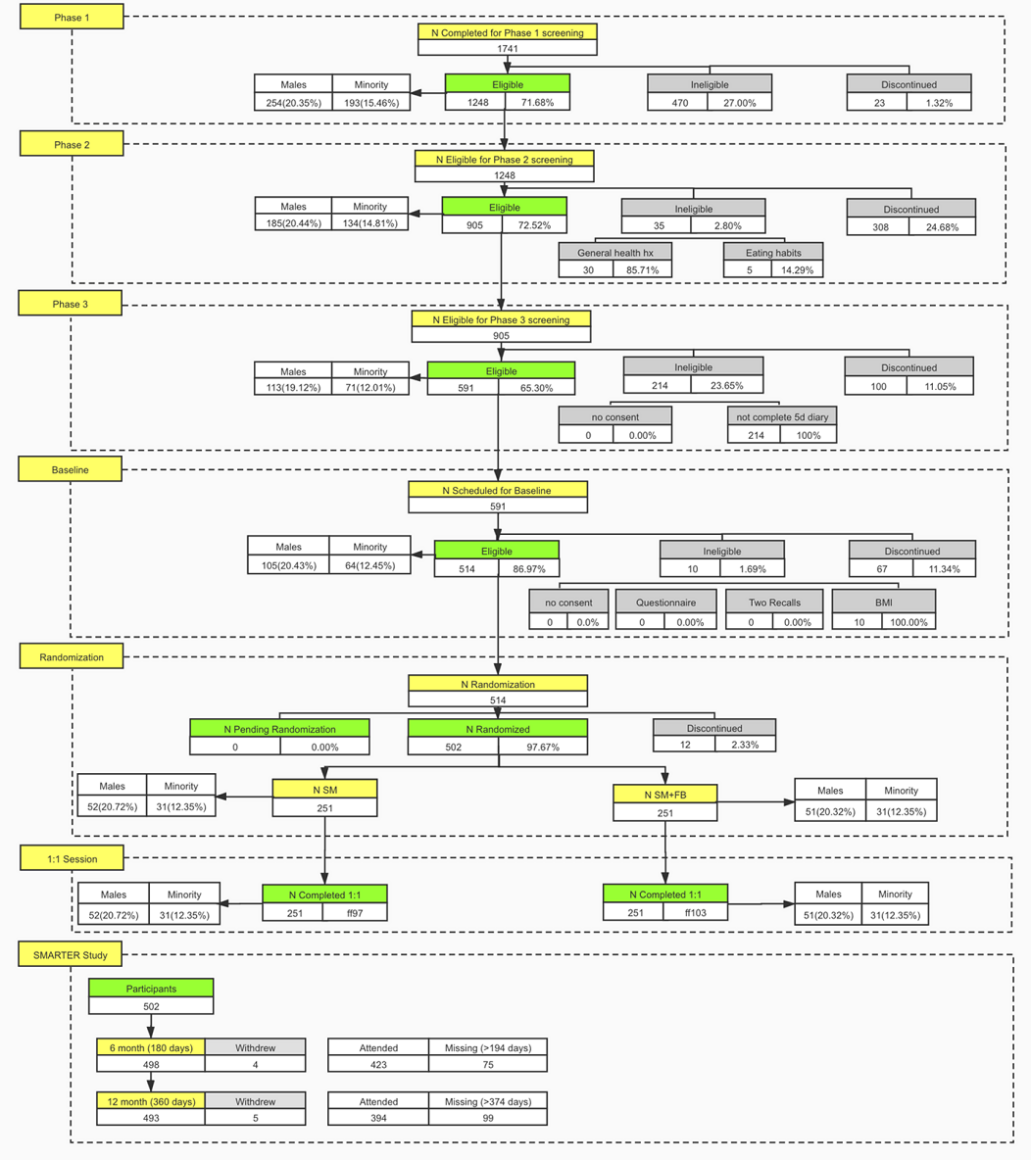
Real-time study flowchart. FB: feedback; SM: self-monitoring.

The ADAM portal was also used for participants’ compensation and reimbursement management. For example, once the payment was made, study coordinators recorded this on the Adam portal’s remuneration page. Another example of a useful feature provided by the ADAM portal for the trial is a real-time flowchart (Figure 5). The flowchart provided an overview of the entire study at any given time, which allowed for an adaptive recruitment strategy and effective adjustments to study resource allocation as needed. Furthermore, using the ADAM portal, the research staff coordinated activities with each other via a single platform remotely, since everything related to the study was accessible in real time and on the web.

### SMARTER App

**Figure 6.**
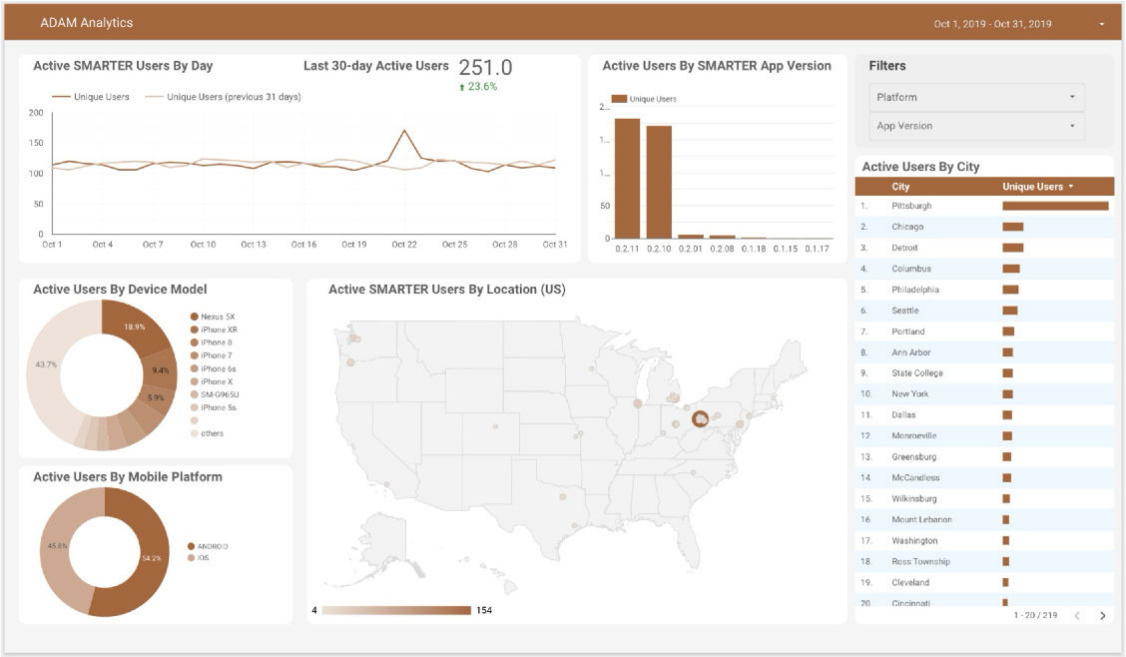
SMARTER app use. ADAM: Awesome Data Acquisition Method.

**Figure 7.**
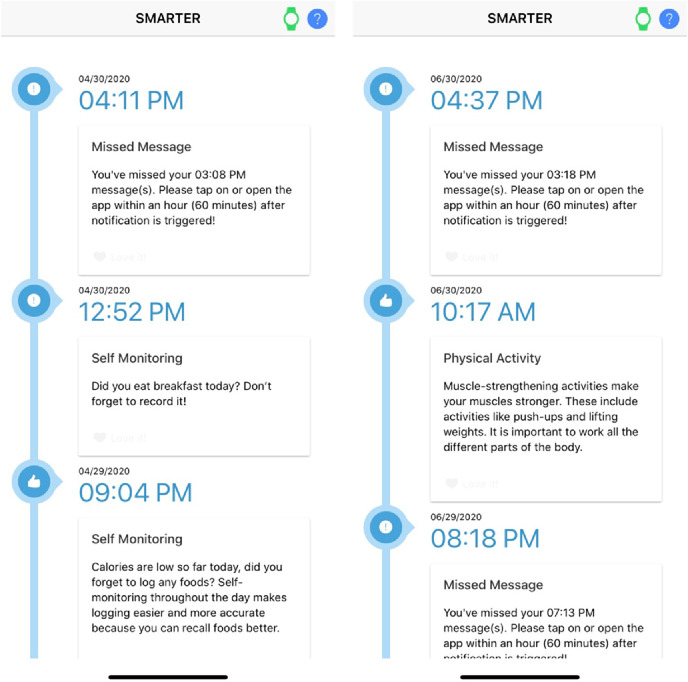
Screenshots of the SMARTER app.

The SMARTER app is an ADAM-integrated app that was designed for the SMARTER trial. Only the intervention group had access to the app. Figure 6 shows the SMARTER app use data based on reports provided by Google’s Firebase Analytics, which included aggregated data for the following aspects: number of daily active users, popular app versions, most common mobile devices among participants, and active users by city. This report worked as supporting information for the study team to track app use. As for the app’s content, Figure 7 shows sample messages tailored and delivered by the SMARTER app. The ADAM system tracked and logged these messages’ time stamps and status on whether the messages were delivered, opened, or missed—information for future data analysis that may provide auxiliary insight into the overall outcome of the trial.

### Data Validation and Integrity

The ADAM system incorporated 4 monitoring mechanisms to ensure the quality of collected data. First, as illustrated in Figure 4B on the clinical dashboard, ADAM can generate alerts. To guarantee data quality from various sources, the system sends automatic alerts for the following reasons: (1) if there are no food data recorded for 7 consecutive days; (2) if no weight data are recorded for 7 days or if there is a recorded weight change exceeding 5 lbs within a 7-day period; and (3) if there are no Fitbit tracker data received for 7 consecutive days. Second, the system performed automatic checks on API connections and promptly reported any issues to the study team. Third, to further ensure data integrity, study coordinators manually checked and reviewed selected records during the in-person assessments. Fourth, before data analysis, the research team also ran an algorithm to check for outliers in the collected data.

## The SMARTER Trial Evaluation Results

In the SMARTER trial, 1741 unique individuals submitted their phase-1 survey through the ADAM portal; 1248 participants were eligible for the phase-2 screening; 905 participants were eligible for the phase-3 screening; and 591 participants were eligible for the baseline in-person assessment. After the baseline assessment, 502 eligible participants were randomized to 1 of the 2 treatment conditions. During the 12-month study period, 4 participants officially withdrew before their 6-month assessment, and 5 withdrew before the final 12-month assessment, all due to health issues. Even including the COVID-19 pandemic, the overall retention rate for the 6-month assessment was 84.93% (423/498) and for the 12-month assessment was 79.92% (394/493). The ADAM system efficiently coordinated study information and supported the study and study team, especially in a situation of limited human resources and having to work fully remotely. It helped to recruit, screen, maintain, and track all individuals wishing to participate in the study; scheduled more than 1500 in-person assessments; and received 4131 questionnaire answers from the screening process to the study completion. Using the ADAM system allowed us to achieve every milestone in the study and minimize the disruptions by the COVID-19 pandemic.

## Discussion

### Principal Findings

We introduced the architecture and implementation of a data collecting and clinical trial management system called ADAM as a solution to current barriers in clinical or behavioral studies involving wearables, IoT, and mHealth. The ADAM system’s adoption of the newest available interfaces in integration with multiple wearable devices makes it an attractive choice for behavioral and population-based health intervention studies. Using the ADAM system, the study team in the SMARTER trial no longer needed to switch between different systems or documents when working with study data from diverse sources and avoided most data missingness and data synchronization issues. ADAM’s study management functions supported recruitment, screening, and randomization and helped maintain retention with a dashboard and flagging. In addition, it provided an efficient and manageable environment for study tracking and adjustment as necessary. ADAM’s study flowchart was extensively used in the team’s weekly meetings and contributed widely to decision-making during the entire study period.

### Limitations

The system evaluations throughout the SMARTER trial indicated that the ADAM system was able to meet most of the data collection and study management demands. However, the ADAM system was not without limitations. The first limitation was not due to the ADAM system per se, but due to the limited availability of open APIs for third-party data collection sources also used in the study; therefore, the ADAM system had issues including all datasets for the study. For example, in the SMARTER trial, study participants needed to complete two 24-hour dietary recalls using the ASA24 system, a dietary assessment tool, at 3 assessment points. We could not directly synchronize the dietary recalls from the ASA24 system with the ADAM system due to the API limitations of the ASA24 platform. This limitation prevented study coordinators from receiving real-time data visualization and data analysis in the ADAM system for ASA24’s data. Therefore, all the data from ASA24 needed to be manually downloaded and uploaded to the ADAM system. Although the ADAM system can integrate more datasets and be an all-in-one data solution for the study, we still need to consider approaches that may have API restrictions or the unavailability of such APIs by different companies and organizations that provide additional data required by the study.

The second limitation to consider is the data missingness and data synchronization issues with Fitbit or Nokia devices. Fitbit’s open web API limits the number of API calls per hour for each participant or Fitbit account to 100. This stringent quota restricted the number of days that we could pull the data from the Fitbit server, as we needed to have at least a single call for each day of data. Furthermore, specifically for the SMARTER trial, we were restricted to pulling data only for the last 7 days. Since we were pulling data from several different areas of Fitbit’s API, such as physical activity data, diary data, and sleep data, it quickly consumed the quota for the API calls. At the same time, from time to time, some Fitbit and Nokia devices experienced issues sending data to the Fitbit server when there were internet or Bluetooth connection problems or participants failed to open the Fitbit or HealthMate app for an extended amount of time. Those situations caused a data transfer delay and ultimately led to data missingness and failed data synchronization in the ADAM system.

Furthermore, the version of ADAM used in the SMARTER trial did not have the capabilities to send emails, SMS text messages, or push notifications. Therefore, the interventionists and study coordinators needed to use different modalities of communication and tools to send emails or SMS text messages to the participants. The next iteration of ADAM will include these capabilities to streamline communications and monitoring within one system, ADAM. This would increase the efficiency and the general ease of using the system in reaching participants as needed.

Finally, the ADAM system is more suitable for digital intervention studies using consumer technologies. If the study is highly dependent on in-clinic assessments using offline tools and closed or proprietary systems, the ADAM system provides a limited benefit since the data may be hard or even impossible to transmit to the ADAM system. In addition, studies that target recruitment groups who are unfamiliar with mobile technology or who have limited access to the internet could experience barriers to the adoption of the ADAM system technology. According to the questionnaires, these groups represent a small portion of potential study participants, but this is an important limitation to consider in future studies that may target these groups. This limitation was not an issue in the SMARTER trial, as the study was designed to exclude participants who were not smartphone users.

To address these limitations, first, we are considering supporting additional, widely used wearable devices. Second, we plan to generalize the study management components and make them easier to set up and adapt to distinct types of study design. However, overall, the ADAM system provides a solution to digitalizing a research study by providing and integrating multiple methods for collecting study data and ensuring an efficient and compliant study process.

## Abbreviations

ADAM: Awesome Data Acquisition Method
API: application programming interface
CONSORT: Consolidated Standards of Reporting Trials
HR: heart rate
IoT: Internet of Things
mHealth: mobile health
SM: self-monitoring
